# The implications of age-related neurofunctional compensatory mechanisms in executive function and language processing including the new Temporal Hypothesis for Compensation

**DOI:** 10.3389/fnhum.2015.00221

**Published:** 2015-04-24

**Authors:** Ruben Martins, Yves Joanette, Oury Monchi

**Affiliations:** ^1^Centre de Recherche, Institut Universitaire de Gériatrie de MontréalMontréal, QC, Canada; ^2^Department of Radiology, Faculty of Medicine of the University of MontrealMontréal, QC, Canada; ^3^Department of psychiatry, Faculty of Medicine of McGill UniversityMontreal, QC, Canada; ^4^Departments of Clinical Neurosciences, Radiology, and Hotchkiss Bran Institute, University of CalgaryCalgary, AB, Canada

**Keywords:** language, execution, cognition, aging, fMRI

## Abstract

As the passage of time structurally alters one’s brain, cognition does not have to suffer the same faith, at least not to the same extent. Indeed, the existence of age-related compensatory mechanisms allow for some cognitive preservation. This paper attempts to coherently review the existing concepts of neurofunctional compensation when applied to two different cognitive domains, namely executive function and language processing. More precisely, we explore the Cognitive reserve (CR) model in healthy aging as well as its two underlying mechanisms: neural reserve and neural compensation. Furthermore, we review the Compensation-Related Utilization of Neural Circuits Hypothesis as well as the Growing Of Life Differences Explains Normal Aging model. Finally, we propose, based on some functional neuroimaging studies, the existence of another compensatory mechanism characterized by age-related delayed cerebral activation allowing for cognitive performance to be preserved at the expense of speed processing: the Temporal Hypothesis for Compensation.

## Introduction

As years pass by, brain modifications occur: the cerebrum loses 1–2% of its mass each year as well as white matter structural integrity (Raz et al., 1997; Moseley, 2002; Head et al., 2004; Sullivan and Pfefferbaum, 2006; Caserta et al., 2009; Fjell et al., 2009). Actually, it has been widely found that the cerebrum weight declines at a rate of around 5% per decade after age 40 with the actual rate of decline increasing significantly particularly over age 70 (Scahill et al., 2003). Furthermore, a decrease in dendritic synapses or loss of synaptic plasticity has also been described (Barnes, 2003). Those changes in integrity and volume appear to be particularly important in the prefrontal cortex, the striatum and the hippocampus, regions of primordial significance in executive function and memory (Raz, 2004). But, do age-related brain changes affect different cognitive domains in the same manner? Do language and executive processes (Table 1) rely on the same compensatory mechanisms to maintain performance as one gets older? Surprisingly, not many studies have tried to answer this question, possibly because it is somewhat difficult to separate pure language processing from executive function which is often required during the execution of language tasks (Just et al., 1996; Stromswold et al., 1996; Van der Linden et al., 1999; Kemper and Sumner, 2001; Humphries et al., 2006). Furthermore, findings tend to be inconsistent. Indeed, while some studies have reported little age-related performance decline (Burke et al., 2000; Waters and Caplan, 2005; Burke and Shafto, 2008) in language abilities, others have shown that older individuals may display impaired execution during language production tasks (Ivnik et al., 1996; Bóna, 2014), more errors when accessing phonological word forms (Shafto et al., 2007), decreased speech comprehension (Schneider et al., 2005) and perception not related with hearing loss (Bilodeau-Mercure et al., 2015) as well as more tip-to-the-tongue states (White and Abrams, 2002). However, it appears that at least some of these findings could be explained by a decline in working memory instead of actual language processing *per se* (Waters and Caplan, 2005).

**Table 1:** **Cognitive domains**.

Concepts	Definitions
Working memory	Process that allows to maintain and to immediately manipulate available information
Executive processing/function	Cognitive processes that support strategic organization and control other processes that play an important in complex, goal oriented tasks
Language processing/abilities	Multiple cognitive processes allowing for the processing of grammatical rule which interplays with phonology and semantics

Enlightened by this last statement, one could then argue that there is less age-related decline in language processing compared to working memory and executive processing. However, even for executive function, it has been shown in some experiments that the age-related decrease in performance would disappear if non-cognitive components (such as motor-speed) were accounted for Fristoe et al. (1997) and Parkin and Java (1999). Some studies have even suggested that there is no age-related executive decline at all (Boone et al., 1990). Moreover, other cognitive domains, such as semantic knowledge (Craik and Jennings, 1992; Park et al., 2002; Verhaeghen, 2003; Burke and Shafto, 2008; Laver, 2009) and emotional regulation (Carstensen et al., 2003, 2011), are clearly maintained with age.

Consequently, given the fact that for some healthy old individuals, cognition appears to be largely maintained (semantic knowledge, emotional regulation, etc.) or less impaired than age-related brain atrophy would suggest (language and executive processing), the aim of the present review is to explore the compensatory mechanisms that would allow for this preservation to occur. Furthermore, the present review will explore if these compensatory mechanisms are the same for executive functions (including working memory) and language processing, or if different cognitive domains rely on different mechanisms.

## Cognitive Domains

Executive function can be summarized as the general cognitive processes that support strategic organization and control other processes that play an important role in complex, goal oriented tasks (Buckner, 2004; Table 1). Working memory, on the other hand, allows to maintain and to immediately manipulate available information. It relies on brain systems that represent memories in an active, online form (Buckner, 2004; Table 1). Based on those definitions, one can see how those two concepts, namely executive function (or processing) and working memory, are intimately related, especially when it comes to the manipulation of information in order to achieve a goal. For the purpose of this review, working memory and executive processing will therefore be considered as a single cognitive domain.

Language abilities can be considered as involving multiple cognitive processes allowing for the processing of grammatical rule which interplays with phonology (the speech sound processing system) and semantics (the meaning processing system) (Hauser et al., 2002; Table 1). Those three systems, themselves composed of several subsystems, enable us to create and understand a potentially infinite number of sentences by using various combinations of words. Even if this review considers, for sake of simplicity, language abilities and executive function as different sets of processes, one should be aware that in reality (and that includes the performance of language processing tasks) those cognitive domains are almost always intimately linked an cannot be easily separated (since working memory, for example, is often needed during the manipulation of language attributes—sounds, works, sentences). One should, therefore, consider language and executive function not as two completely detached cognitive entities, but as two different domains relying on dissimilar as well as similar brain regions and cognitive processes.

## Cognitive Reserve

**Figure 1:**
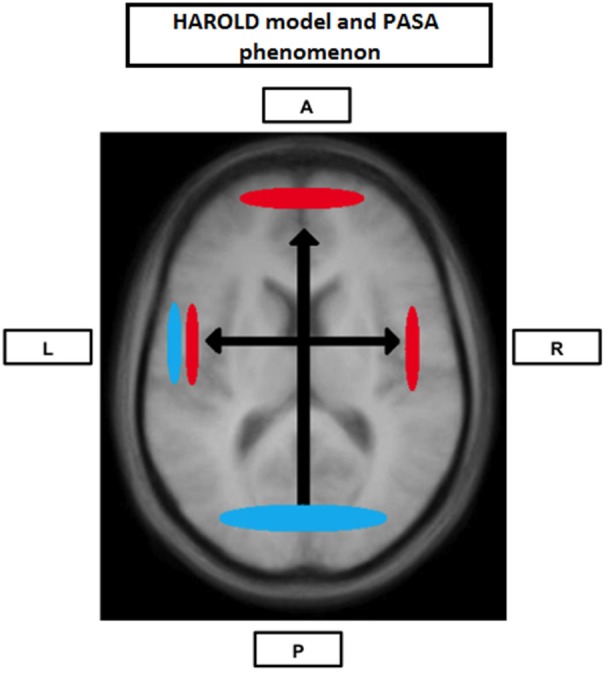
**Neural Compensation**. Brain image illustrating an age-related hemispheric asymmetry reduction in brain activity (HAROLD) and a posterior-anterior brain activity shift (PASA), two phenomena believed to represent age-related neural compensatory mechanisms. Blue represents activity in younger individuals and red represents activity in older individuals. A: Anterior; P: Posterior; L: Left hemisphere; R: Right hemisphere.

The cognitive reserve (CR) hypothesis is a ‘functional’ model of reserve conceptualized by Stern (2002) that reflects the inter-individual ability to effectively use cognitive processes and brain networks (Table 2). Regarding elderly individuals, two CR mechanisms have been proposed: neural compensation and neural reserve (Stern, 2009). Neural compensation is the use of new, compensatory brain networks after pathology or normal aging disrupted those typically recruited for a particular task (Table 2). The neural compensation hypothesis was in part based on the fact that several episodic memory, semantic memory, working memory, perception and inhibitory control task studies have reported that high performing older individuals tended to show bilateralization of cerebral activation (Reuter-Lorenz et al., 2000; Cabeza, 2002; Reuter-Lorenz, 2002; Reuter-Lorenz and Lustig, 2005; Reuter-Lorenz and Park, 2010) as well as intrahemispheric changes in activation patterns, mainly from the occipitotemporal to the frontal cortex (Grady et al., 1994, 2005; Madden et al., 1997; Reuter-Lorenz et al., 2000; Cabeza, 2004; Cappell et al., 2010). These findings led, respectively, to the proposition of the HAROLD (Hemispheric Asymmetry Reduction in OLDer adults) model by Cabeza (2002) and the PASA (posterior-anterior shift in aging) phenomenon by Dennis and Cabeza (2008) (Figure 1). The HAROLD model states that, under similar circumstances, prefrontal activity during cognitive performances tends to be less lateralized in older adults than in younger individuals, it is believed that this “delateralization” has a compensatory function and reflects regional or network mechanisms (Cabeza, 2002). The PASA phenomenon, additionally, has also been shown to reflect the effects of aging (and not differences in task difficulty for example), furthermore age-related increases in frontal activity have been positively correlated with cognitive performance and negatively correlated with the age-related occipital decreases (Davis et al., 2008). Therefore, as previously stated, these patterns of brain activity reorganization may represent a compensatory mechanism based on the recruitment of new brain networks in order to maintain performance. Neural reserve, on the other hand, is another strategy used by healthy individuals when coping with task demands. It emphasizes pre-existing differences in neural efficiency or capacity. It consists in using flexible brain networks or cognitive resources that are less susceptible to disruption (Stern, 2009; Table 2).

**Table 2:** **Compensatory mechanisms**.

Concepts	Definitions
Cognitive Reserve	Inter-individual ability to effectively use cognitive processes and brain networks
Neural Compensation	The ability to use new, compensatory brain networks after pathology or normal aging disrupted those typically recruited for a particular task
Neural Reserve	The ability to use flexible brain networks or cognitive resources that are less susceptible to disruption
Compensation-Related Utilization of Neural Circuits Hypothesis	Individuals activate more cortical regions as task load increases. Older individuals need to engage more neural resources at lower levels than younger adults
Growing of Life Differences Explains Normal Aging	Normal aging represents maturational processes causing progressive shifts in the distributions of mental abilities over the lifespan. Therefore, brain phenomena observed in older individuals are already apparent in younger individuals under appropriate conditions (high-demand cognitive processes).
Temporal Hypothesis for Compensation	Age-related delay in brain activity, particularly in the PFC, during cognitive processing. Age-related shift from proactive to reactive cognitive control strategies when cognitive processes imply both anticipation and resolution. These age-related temporally based functional changes in brain activation patterns allow for cognitive performance to be preserved at the expense of speed processing.

### Neural Compensation

#### Executive Function

Stern and companions have extensively explored the neural compensation hypothesis in several of their studies. Zarahn et al. (2007), part of the same group, published a functional magnetic resonance imaging (fMRI) study in which young and old participants were compared while performing the letter Sternberg task (a working memory task) using Multivariate Linear Modeling (MLM). Their results showed that load-related activation during the retention phase of the task was characterized by two spatial patterns: one composed of areas often associated with working memory (including the cerebellum, the insula, the inferior and middle frontal gyrus, the hippocampus, the superior frontal gyrus, the inferior and superior parietal lobules and cingulate), and another composed only of the right hippocampal region. While the first pattern was used by both the young and the elderly, the second one was only used by the older subjects. Interestingly, the activation of the second network was linked with a decrease in performance.

There are two possible explanations for this finding. One is that this observation cannot represent a compensatory mechanism since as older individuals increasingly use the alternative network, worse is their performance. However, one could also argue that this alternative network is needed to maintain function as age-related neural changes diminish the efficacy of the first (primary) network. In other words, those individuals using the primary and alternative network would perform even worse if they relied only on their (impaired) primary pathway. If the latter explanation proves to be true, the second network would then be an example of neural compensation.

The same group (Steffener et al., 2009) tried to shed some light on this dilemma. They predicted that, if the second network was compensatory, individuals who express the second pathway should have age-related neural changes that affect the primary network. To explore their hypothesis, they used voxel based morphometry (VBM) to test if atrophy in the primary pathway was related to expression of the secondary network, and they found that a decrease in gray matter density of the left pre-central gyrus was linked with an increase in secondary pathway recruitment. They also found that there was a correlation between gray matter density in the pre-central gyrus and age, but only in the elderly. Based on those findings, they postulated that the elderly increasingly recruit alternate pathways when the primary networks are affected by age-related atrophy. Therefore, this is an example of neural compensation in which older individuals use an alternate network to maintain (at a lower level) task performance.

As previously mentioned, several studies exploring different cognitive modalities have obtained results compatible with neural compensation. Among the results more frequently reported is the tendency for high performing older individuals to show interhemispheric dedifferentiation of cerebral activation (Reuter-Lorenz et al., 2000; Cabeza, 2002; Reuter-Lorenz, 2002; Reuter-Lorenz and Lustig, 2005; Reuter-Lorenz and Cappell, 2008; Reuter-Lorenz and Park, 2010) and intrahemispheric changes in activation patterns, mainly from the occipitotemporal to the frontal cortex (Grady et al., 1994, 1998, 2005; Madden et al., 1997; Reuter-Lorenz et al., 2000; Cabeza, 2004; Cappell et al., 2010): the HAROLD model and the PASA phenomenon (Figure 1).

Recently, Springer et al. (2005) have shown using a working memory task that high performing old individuals tend to rely more extensively on frontal regions and that those regions tend to be bilaterally activated. This observation is in agreement with both the HAROLD and PASA phenomena. However, their complementary analysis did not show any significant correlation within old participants either between frontal activity and performance, or between frontal activity and level of education. Based on those findings, it is difficult to argue for or against the compensatory nature of this increase in frontal recruitment. However, other studies looking at aging and executive processes have shown an increase in frontal activity in older individuals compared to younger persons. Indeed, our group (Martins et al., 2012) has found that high performing older individuals tended to rely more extensively than younger individuals on the frontopolar cortex while performing a lexical version of the Wisconsin Card Sorting Test (WCST—a test often used by neuropsychologists to assess executive function). In this version of the WCST, also known as the Wisconsin Word Sorting Task—WWST (Simard et al., 2011), participants have to match test words with reference words according to one of the following rules: syllable onset, syllable rhyme or semantics. Participants are unaware of which rule they have to apply and have to find it by trial and error (as for the classical WCST). In that particular experiment, the difference in performance between the younger and older group was minimal. Moreover, it should be noted that, in some studies in which the elderly presented impaired performance, age-related decreased frontal activity has also been reported both in PET (e.g., Reuter-Lorenz and Cappell, 2008) and fMRI studies (e.g., Hampshire et al., 2008) suggesting that increased frontal and bilateral activity are indeed neuronal compensation mechanisms.

Several studies using functional near infrared spectroscopy (fNIRS), which measures the hemodynamic correlates of neural activity, have also shown how increased right DLPFC activity is often associated with better performance during executive tasks in healthy older adults. For example, Albinet et al. (2014) investigated two groups of women aged from 60 to 77 years old divided accordingly to their cardiorespiratory fitness (high and low). Their results showed that during the performance of a random number generation (RNG) task, the high-fit women were able to generate sequences with less adjacent numbers than the low-fit group. More interestingly, not only did high-fit women perform better, they also showed significant increases in oxyhemoglobine responses in both the left and right DLPFC during the task, while the low-fit women showed significantly less activation in the right DLPFC compared with the right DLPFC of the high-fit group and compared with their own left DLPFC. Another study (Vermeij et al., 2014) using fNIRS and a working memory task (the spatial 1 low load—or 2 high load—back task) also showed that high performers activated the right PFC under high working-memory load conditions more strongly than low performers did. Furthermore, Müller et al. (2014) found that older individuals tended to be slower, but also made less mistakes than younger individuals in a trail making test. Those results were associated with increased bilateral activity in the DLPFC for the elderly, a pattern that the authors related to the HAROLD model. Another study (Hagen et al., 2014) using fNIRS and a trail making test showed that older individuals had less focused prefrontal activation than younger participants. Those results could represent the recruitment of new networks by the elderly, and therefore, neural compensation. Finally, Heilbronner and Münte (2013) revealed that older participants (over 60 years old) relied more on the right DLPFC than younger individuals (below 30 years old) during a simple visual Go/NoGO task, another result compatible with the HAROLD model.

#### Language Abilities

Several neuroimaging studies that looked at language processing have also reported increased bilateral activity in high performing older persons compared with younger individuals during verbal generation (Persson et al., 2004) and naming tasks (Wierenga et al., 2008). More recently, Obler et al. (2010) have even shown anatomical evidence (using diffusion tensor imaging) that older individuals with high naming skills tended to rely more extensively on right-hemisphere frontal regions (peri-Sylvian and the midfrontal areas). Therefore, those results seem to indicate that language function also relies on neural compensation.

In 2002, Grossman et al. (2002a) published an article in which brain activity of young, older good and older poor “performers” was compared while the participants were performing a language task. The task consisted in answering a probe question about who performed the action described in a sentence previously presented. The older good performers were as accurate as the younger participants, while the older poor performers showed impaired sentence comprehension compared to the young individuals. The difference between the poor performers and the other two groups of participants became more important as sentences became more syntactically complex.

Regarding brain activity patterns, older good performers showed significant increased activation in two areas compared to their younger peers. Indeed, the dorsal portion of the left inferior frontal cortex (IFC), an area known to play a role in working memory including maintaining and rehearsing stored verbal information (Smith et al., 1998; Chein and Fiez, 2001), was more activated in the older group. Moreover, the successful older adults also showed additional activation in the right posterolateral temporal-parietal region (while the left counterpart was more activated in the younger group). Those two findings are in agreement with neural compensation, and they seem to show the co-occurrence of the PASA phenomenon and the HAROLD model.

When the activation pattern of the poor performers was compared to the one of the good performers, it was revealed that the poor performers had increased dorsolateral prefrontal cortex (PFC) activity. The dorsolateral PFC has been reported in several studies implying problem-solving activities, regardless of the nature of the material (e.g., Monchi et al., 2001; Paulus et al., 2001; Ramnani and Owen, 2004; Simard et al., 2011; Martins et al., 2012). That region was not activated in the younger group. Therefore, it seems that the less successful older participants were attempting to understand more grammatically complex sentences by using a problem-solving approach that was not very effective for this particular task. This finding may as well be another example of neural compensation in which poor performers recruit the dorsolateral PFC in an attempt to compensate for age-related insults; unfortunately, contrarily to the good performers, their “strategy” is not sufficient to adequately perform during sentence comprehension.

Tyler et al. (2010) explored syntactic processing in older individuals and found that bilateral recruitment of frontotemporal regions was correlated with improved performance. More recently, Ansado et al. (2013) studied the comprehension of word semantics using a semantic judgment task. During the fMRI experiment, young and old participants had to indicate if a given word presented on a screen identified an animal or not. Behavioral results were similar for both groups, with slightly longer response times for the older one. The fMRI results, on the other hand, showed that older individuals had more parietal and temporal bilateral activations as well as left fusiform activations, while younger subjects had more dorsolateral PFC activations. In the same article, Ansado et al. (2013) also presented data from another preliminary study in which young and older healthy individuals had to perform a verbal fluency (VF) task which involved eight alternating 90-s blocs of four orthographic and four semantic VF conditions as well as a reference condition (repeating the months of the year). The neuroimaging results showed that older individuals had increased bilateral temporal activations during semantic conditions, while similar frontal activations were observed in both groups. However, older participants showed more frontal bilateral activations during orthographic conditions. Both experiments showed that the elderly had a pattern of activation compatible with the HAROLD model (increased bilateral activity in the elderly). However, the apparent posteriorization of some activation in the older group (during semantic judgment and semantic fluency) is in contradiction with the PASA phenomenon.

The authors mention that the discrepancy between their results and what is usually shown in the literature may suggest that during the semantic judgment task, older individuals rely more on their semantic memory and knowledge (processes more associated with posterior regions) while younger individuals rely more on an executive strategy (which imply the involvement of the PFC). They also point out that semantic fluency tends to rely on temporal regions whereas orthographic fluency is more dependent on frontal regions (Henry and Crawford, 2004) which would explain the results of the VF experiment. These observations are actually congruent with other semantic neuroimaging studies (Hazlett et al., 1998; Wingfield and Grossman, 2006) in which older participants presented increased posterior activation. Therefore, Ansado et al. (2013) propose that the nature of a task seems to be a determinant factor for neurofunctional activity reorganization in aging. Even if we agree with this conclusion, we also would like to point out that both age-related anteriorisation and posteriorisation of activation are examples of neural compensation. Consequently, different language domains appear to rely on similar compensatory mechanisms, namely neural compensation (even if the form of the neural compensation may vary).

**Figure 2:**
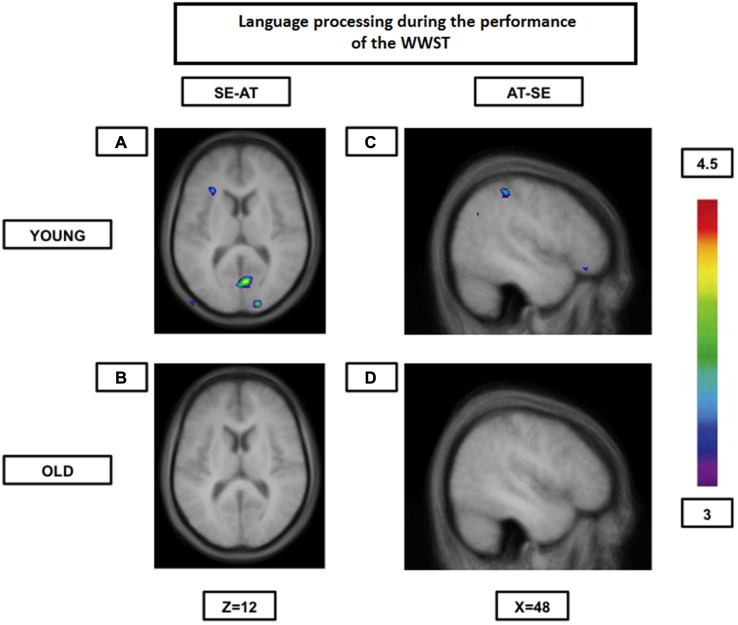
**Brain activation contrasts during language processing using the WWST in young and old adults**. Old individuals show fewer differences in brain activity between different language processes than young individuals. **(A)**: Activation in the left ventrolateral PFC (areas 45 and 47/12) and in right occipital regions (areas 17 and 18) when SE is compared to AT in young adults. **(B)**: No significant peaks of activation at all when SE is compared to AT in old adults. **(C)**: Activation in the right frontopolar area (area 10) and the right posterior parietal cortex (area 40) when AT is compared to SE in young adults. **(D)**: No significant peaks of activation at all when AT is compared to SE in old adults. The anatomical MRI images are the average of the T1 acquisitions of 14 younger subjects and 14 older participants transformed into stereotaxic space. The color scale represents the *T* statistic. SE: correct matching according to semantics events; AT: correct matching according to syllable onset(attack) events.

Our group (Martins et al., 2014) has used the WWST to study how aging affected brain patterns involved in semantic and phonological (syllable onset and rhyme) processing comparing young to high performing old participants. In this particular analysis, only accurate matching trials were considered. Our results indicated that while young adults tended to show increased activity in the ventrolateral PFC, the dorsolateral PFC, the fusiform gyrus, the ventral temporal lobe and the caudate nucleus during semantic decisions (semantic pathway) and in the posterior Broca’s area, the temporoparietal junction and the motor cortical regions during phonological decisions (phonological pathway), older individuals showed increased activity in regions of the two pathways during both semantic and phonological decisions. Therefore, in older individuals, the semantic and phonological routes seemed to merge into a single route composed of the semantic and the phonological pathways. This was even more evident when brain activity during one rule was contrasted to the activity during another rule: young individuals showed significant inter-rule activity differences, while the elderly did not (Figure 2). These findings represent, once again, most probably a neural compensation mechanism on which the elderly rely to maintain an adequate level of performance.

Similar neural compensatory mechanisms have also been found during speech comprehension tasks. Indeed, age-related hearing loss is accompanied by auditory cortex atrophies (Harris et al., 2009; Eckert et al., 2012; Peelle et al., 2013), explaining why older adults likely have to recruit different neural resources in order to maintain appropriate speech comprehension. That explains why Eckert et al. (2008) observed an age-related upregulation of frontal areas during an easy word recognition task in older individuals, while younger adults recruited these areas merely during difficult listening conditions. Wong et al. (2009) also found that during single word recognition tasks older subjects, when compared to younger ones, showed reduced activation in the auditory cortex but an increase in working memory and attention-related cortical areas (prefrontal regions). Both of these findings are compatible with the PASA phenomenon. More recently, Erb and Obleser (2013) studied neural speech processing in a group of older adults with varying degrees of sensorineural hearing loss and a group of younger individuals with normal hearing. All the subjects had to hear and repeat back degraded sentences. Their results showed that the older adults adapted to degraded speech at the same rate as younger listeners; however, for correct speech comprehension, older individuals relied on the middle frontal gyrus in addition to a core speech comprehension network recruited by the young which is suggestive of a compensatory mechanism.

In 2014, Amiri et al. (2014) investigated younger (aged 20–35) and older (aged 65–75) individuals the participants were presented with letter strings (4–6 letters) and had to answer whether or not the strings constituted real words or a pseudo-words. Using fNIRS (with structural MRI and baseline physiological assessments), they observed more significant age-related activity around the junction of the inferior frontal gyrus and inferior precentral sulcus, along with engagement of Wernicke’s area, all in the right hemisphere during lexical-semantic processing. These differences in hemispheric patterns of activity could represent new network recruitment by the older group in the right hemisphere, meaning that activation might have become less lateralized with aging in agreement with the HAROLD model. Heinzel et al. (2013) have also presented results compatible with neural compensation. Indeed, using fNIRS and a VF (phonological and semantic) task, they showed that age predicted reduced inferior frontal junction and increased middle frontal and supramarginal gyri activity for both task components. In other words, the elderly might have relied on different networks than younger participants in order to maintain task performance. Another study (Scherer et al., 2012) compared the brain activity of ten young with ten old individuals during the performance of a narrative discourse task. Their results showed several differences between the two groups both within- and across-hemispheres which the authors interpreted as manifestations of neural compensation. Finally, using NIRS, Herrmann et al. (2006) showed that older individuals presented no left hemispheric lateralization effect contrarily to younger participants during the performance of a VF task: results compatible with the HAROLD model.

#### Are Phenomenon such as PASA and HAROLD Necessarily Compensatory?

Most of the studies presented in this review rely on functional neuroimaging to “measure” brain activity. However, one has to be careful when interpreting fMRI data. While it is appropriate to consider BOLD signals as measures of neural activity of a specific brain region in healthy young adults, the validity of such interpretations is less robust when comparing signals across individuals or states during which significant variations in physiology may prevail. Indeed, increasing evidence suggests that changes in neurovascular coupling (due to medication, disease, age, etc.) have the potential to significantly modify task-related BOLD responses (Carusone et al., 2002; D’Esposito et al., 2003; Iannetti and Wise, 2007; Lindauer et al., 2010; see Liu, 2013 for review). Therefore, the PASA phenomenon, for example, may as well represent age-related changes in patterns of brain activity as changes in vascularity.

Secondly, age-related over-recruitment, particularly bilateralization of cerebral activity, has been interpreted as compensatory both when the correlation between bilateral activity and performance was positive (Persson et al., 2004; Springer et al., 2005; Wierenga et al., 2008; Obler et al., 2010), as well as negative (Steffener et al., 2009; de Chastelaine et al., 2011). Indeed, as previously mentioned, Steffener et al. (2009) postulated that increased recruitment of the right hippocampal region by the elderly (Zarahn et al., 2007) during the performance of a working-memory task was compensatory even if the overall performance was worse in the older group compared to the younger one. de Chastelaine et al. (2011) also found, during a verbal encoding memory task, that increased right frontal activity in older adults was negatively correlated with memory performance, and they too postulated that this increased right hemisphere recruitment could nonetheless reflect the engagement of processes that compensate only partially for age-related neural degradation, therefore the impaired performance. Cabeza and Dennis (2012) expanded on this idea and hypothesized the existence of three different types of compensation: “attempted”, “unsucessful”, and “successful” compensation. When there is a mismatch between available cognitive resources and task demands, additional neural resources are recruited, reflected in increased brain activity. This over-recruitment is called “attempted compensation”. If the increase in brain activity is associated with better task performance, it then becomes an example of “successful” neural compensation. On the other hand, if it is associated with worse task performance (as for the examples presented above), it is then defined as “unsuccessful” neural compensation. In the fNIRS study of Vermeij et al. (2014) presented earlier, older high performing individuals showed increased activity in the right PFC during the high-load condition of a working memory task compared to the age-matched low performing subjects. However, within low performers, the authors found that those who demonstrated a larger load-induced decline in behavioral performance showed a larger load-induced increase in bilateral PFC activation. Therefore, Vermeij and colleagues postulated this load-induced increase in some low performers could represent “unsuccessful” neural compensation as hypothesized by Cabeza and Dennis (2012).

Another possible explanation for increased brain activity in the elderly is that it does not represent any type of compensation at all, but is actually a manifestation of age-relate brain disruption. Such hypothesis has been favored in some studies in which over-recruitment was associated with impaired cognition (e.g., Duverne et al., 2009). With age, one would lose the ability to inhibit certain regions of the brain, those areas would therefore be more activated in older individuals during the performance of a cognitive task, but they would not contribute to cognition. Differentiating “unsuccessful compensation” from “disrupted over-activation” is almost impossible, especially since both mechanisms can most probably concomitantly occur.

We have mentioned earlier that certain cognitive domains don’t appear to show any performance decline with aging (e.g., emotional regulation) (Carstensen et al., 2003, 2011), some may even show improvement, such as semantic knowledge (Craik and Jennings, 1992; Park et al., 2002; Verhaeghen, 2003; Burke and Shafto, 2008; Laver, 2009). Thus, is it appropriate to talk about “compensation” when performance improves? Therefore, we would like to offer yet one more possible explanation for increased brain activity in the elderly regarding those particular cases, and that is the ability for older individuals to rely on neural over-recruitment, not as means of compensation, but as a “strategy” to increase cognitive performance.

In the present review, the PASA phenomenon, age-related activity dedifferentiation or bilateralization and general neural over-recruitment have been considered manifestations of neural compensation. However, one should keep in mind that this is just one possible interpretation; age-related over-activation could also be a sign, as stated above, of dysfunction (the inability to inhibit certain brain areas) especially when it is correlated with impaired performance; or, on the contrary, improved function when it is associated with better performance.

### Neural Reserve

#### Executive Function

Neural reserve has been extensively studied in the context of working memory. Indeed, Zarahn et al. (2007) scanned young and old individuals while performing the letter Sternberg task, a task involving the presentation of a list of letters to memorize (stimulus phase), followed by a period during which the participants must maintain the list in memory (maintenance phase), because afterwards they are asked to respond if new letters presented to them were in the list they had to memorize or not (probe phase). In that study, it was determined that both the younger and the older groups showed similar spatial patterns during the stimulus and probe phases of the task. The authors decided to address the question of whether there were age-related differences in network efficiency between the two groups as they both showed similar pattern activation. Interestingly, they found that as the task got more difficult, the elderly increased network recruitment to a greater extent in the stimulus phase than the younger participants; however they also benefited less from the network recruitment in terms of performance (they made more errors in the probe phase). This result seems to show how age-related neural changes may impair network efficiency even when the network itself remains unchanged. This being said, the fact that the older group was capable of activating the networks to the same degree as the younger one demonstrates that neural reserve is a compensatory mechanism on which older individuals may rely.

In 2009, the same group (Holtzer et al., 2009) conducted a similar analysis on data resulting from young and old subjects performing the shape Sternberg task. This task is similar to the letter Sternberg task, but uses shapes as stimuli rather than familiar letters. This last feature is believed to make the task more challenging than its close relative (Holtzer et al., 2009). However, once again, both the young and the elderly used similar brain pathways during the performance of the stimulus and probe phases. But in this case, they found that the “probe phase” network expression was greater in the younger group compared to the older one. In other words, the younger individuals performed better and showed increased expression of the underlying brain network, which suggests a capacity difference between the two age groups (that is a difference in the ability to recruit the network in question). It is quite probable that the use of the shape Sternberg task, which is more demanding than its letter counterpart, explains why both age groups show differences in capacity in that study (Holtzer et al., 2009), but not in the previous one (Zarahn et al., 2007). Indeed, the first study was not challenging enough for either group to reach their capacity potential, while the second one was: the elderly reached their capacity limits before the young. Nonetheless, age-related decline in capacity does not equate with elderly inability to rely on neural reserve as a compensatory mechanism, however, it emphasis the need for the co-occurrence of other compensatory mechanisms if function is to be preserved.

In 2012, our group (Martins et al., 2012) have also shown that young and high performing older individuals tend to use the same frontostriatal loops while performing the WWST, namely a cognitive loop including the ventrolateral PFC (area 47/12), the caudate nucleus and the thalamus involved in the planning of a set-shift, and a motor loop important in the execution of a set-shift that includes the posterior PFC and the putamen. Overall, there was no intergroup difference in activation except in the caudate nucleus (significantly more activated in the young) when we looked at all the periods combined (receiving feedback and matching following feedback). Therefore, not only did the elderly rely on CR, they also showed little decline in capacity.

Recently, Oboshi et al. (2014) elaborated an NIRS study in which 60 young and 60 old healthy adults had to perform 6 repetitions of a working memory task, which included a task period as well as a resting period. The authors analyzed the magnitude of the task-related NIRS responses for each period and compared the results between the two age groups. Their analyses revealed that hemodynamic responses in the PFC during the pre-task period (2nd half of the resting period) were smaller in the elderly than in young adults. Furthermore, there was a positive correlation between hemodynamic changes during the pre-task period in the PFC and correct answer ratios in both groups. Therefore, these findings suggest that more pre-task activation in the anterior PFC was associated with better cognitive performance. In other words, younger individuals with higher capacity levels during the pre-task period were better performers, as were older individuals with less capacity decline (or more neural reserve).

#### Language Abilities

Grossman et al. (2002b) have shown that when both older good and poor performers were compared while performing a sentence-comprehension task (which consisted in reading a sentence and answering a question about who performed the action described in the given sentence), poor performers engaged significantly less activation of some important sentence-processing areas in the left IFC and the left posterior-superior temporal cortex relative to good performers. This finding seems to show that old good performers are able to rely more extensively than old poor performers on some language pathways, therefore using neural reserve as a compensatory mechanism.

In the study of Erb and Obleser (2013) (mentioned above) exploring neural speech processing in a group of older adults with varying degrees of sensorineural hearing loss and a group of younger individuals with normal hearing, the authors found that both groups relied on the left anterior insula when presented with degraded more than clear speech. However, anterior insula recruitment in the older group was dependent on hearing acuity. Therefore, older individuals with less impaired hearing were able to rely more extensively on the left anterior insula similarly to the young which represents an example of neural reserve.

Our group (Martins et al., 2014) explored language function and aging using the WWST. As previously mentioned, our results showed that the elderly tended to use similar regions for both semantic and phonological processing. In other words, the elderly showed both cognitive compensation (by relying on regions often associated with phonological processing during semantic processing for example) and CR (by relying on the semantic pathway during semantic processing on top of the other regions often associated with phonological processing) for both semantic and phonological decisions (Figure 2). However, another experiment comparing semantic and phonological processing has shown that the first tends to rely more extensively on age-related neural reserve than the latter. Indeed, Diaz et al. (2014), have found that when younger and older adults were asked to make semantic and phonological decisions about pictures, the older group was as accurate and efficient as the younger one in the semantic task, but not during the phonological task. Interestingly, both groups also showed increased activity of similar left-hemisphere language regions during semantic decisions, while they presented more bilateral and widespread activations during the phonological task (especially the older group). Therefore, the older adults were able to recruit more efficiently left-hemisphere language regions (neural reserve) during semantic processing than during phonological processing which was associated with better behavioral results.

## Compensation-Related Utilization of Neural Circuits Hypothesis (CRUNCH)

**Figure 3:**
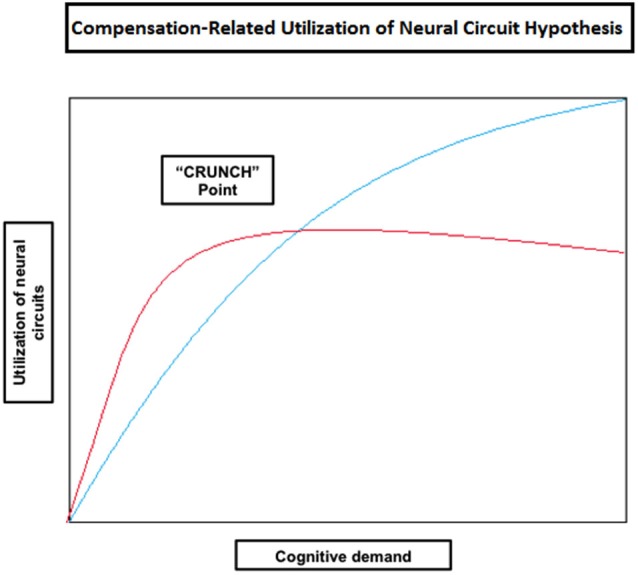
**Theoretical illustration of how neural circuit utilization varies with an increase in cognitive demand in old (red) and young (blue) individuals according to the Compensation-Related Utilization of Neural Circuit Hypothesis**. This model implies that people will generally activate more cortical regions as task load increases; however old individuals need to engage more neural resources at lower levels of cognitive demand than young adults. Old individuals also reach their resource limitations (shown in the figure as the “CRUNCH point”) at lower levels of demand, after which their brain activity may plateau or even decrease as does performance.

Some of the studies reported so far have shown that compensatory mechanisms (brain over-activation) are not limited to older individuals, but they also occur in younger people when task demands increase (e.g., Grady et al., 1998; Rypma and D’Esposito, 2000; Braver et al., 2001a; Logan et al., 2002; Zarahn et al., 2007; Holtzer et al., 2009; Schneider-Garces et al., 2010). Other studies in verbal working memory have also shown that the elderly don’t always reveal increased brain activity compared with the young, but under-activation instead, mainly at the level of the dorsolateral PFC (Rypma and D’Esposito, 2000; Rypma et al., 2001; Ansado et al., 2013). These observations led Reuter-Lorenz and Cappell (2008) and Reuter-Lorenz and Lustig (2005) to propose a model implying that people will generally activate more cortical regions as task load increases (Compensation-Related Utilization of Neural Circuits Hypothesis; CRUNCH) (Table 2). However, due to age-related decline in neural processing and efficiency, older individuals might need to engage more neural resources at lower levels than younger adults. It should be noted that this hypothesis does not enter in conflict with the concept of CR proposed by Stern (2002); on the contrary, it complements it. Indeed, as age advances and CR diminishes, older individuals will need to rely more heavily on task specific pathways (neural reserve) and/or other brain areas (neural compensation) at low task loads. Therefore, it is expected for old individuals to reach their resource limitations (in terms of cortical regions used) at lower levels of cognitive demand compared to younger individuals, leading to a decline in performance as demand increases. At this “crunch” point, brain activity may plateau or even decrease with increasing task loads, explaining why some studies report that the elderly show reduced brain activity compared to the young or higher performing individuals (e.g., Hampshire et al., 2008; Reuter-Lorenz and Cappell, 2008; Figure 3).

### Working Memory

Some studies have been designed to explore the CRUNCH model, especially in the context of working memory. For example, Cappell et al. (2010) scanned (using fMRI) young and old adults while performing a verbal memory task with a load varying between four, five and seven letters. Older adults performed as well as the younger ones when verbal memory loads were of four or five items, but less accurately for memory loads of seven letters. Interestingly, and with agreement of the predictions of the CRUNCH model, the elderly showed brain over-activation when their performance was similar to the young and under-activation with increased memory load and reduced performance (mainly in the right dorsolateral and ventrolateral PFC). Another study performed by Schneider-Garces et al. (2010) showed similar results. Indeed, in that experiment, young and old subjects were scanned while performing the letter Sternberg’s task with memory set sizes varying from two to six letters. The behavioral data indicated that the older group had significantly more difficulty with the task than the younger one, especially when set sizes were larger than four items. On the other hand, the fMRI data showed that several brain regions (including the PFC) had significant bilateral increases of activity as set sizes got larger and thus for both groups. However, while older adults presented a large increase in brain activation between set sizes of two and four letters as well as a negligible further increase at larger set sizes, younger adults showed most of their increase at larger set sizes (five and six letters). Once again, the elderly tended to rely on compensatory mechanisms at lower levels of cognitive demand and reached their resource limitations faster than the young.

Vermeij et al. (2012) have also shown patterns of activity compatible with the CRUNCH model using fNIRS. Indeed, they examined aging-related changes in left and right PFC activation during the performance of a working-memory task by healthy young and old individuals. In both groups, bilateral prefrontal activation increased with rising working-memory load. However, while the young showed a continuous increase of the hemodynamic response during higher working-memory demands, older individuals presented a quadratic pattern of activation. Based on these results, it seems that older adults recruited both hemispheres at lower levels of working-memory load and were reaching their limitations faster compared to the young in accordance with the CRUNCH model.

### Language Abilities

Even if the study of Grossman et al. (2002b) was not designed to explore the CRUNCH model in sentence comprehension, it nonetheless shows quite elegantly how the model may apply to language processing. Indeed, in that study, older poor performers show significantly less activation of some important sentence-processing areas when compared to old good performers, and thus especially when task load increases. In other words, old poor performers have reached their resource limitations while old good performers have not. (Meinzer et al., 2012a,b) have also shown, using fMRI and a VF task during which participants had to generate in a limited amount of time as many words as possible under specific category conditions (e.g., animals), that increased bilateral compensatory activity (especially in the inferior gyrus) was mediated by task difficulty more than by age. In other words, as task demands increased, both the young and the elderly showed more bilateral activations which is congruent with the CRUNCH model. Finally, Eckert et al. (2008), in a study presented earlier, observed an increase in activity in frontal regions during an easy word recognition task in older individuals, while younger adults only recruited these areas during difficult listening conditions. Therefore, over-recruitment was not solely due to aging, but task difficulty as well in accordance with the CRUNCH model.

Scherer et al. (2012), in another fNIRS study mentioned above, found that young and old individuals presented several differences in their activity patterns during the performance of a narrative discourse task. However, a third group composed of younger adults performing the task in a non-proficient second language showed activity patterns closer to those of the older group rather than the younger one. Furthermore, Kahlaoui et al. (2012) found a bilateral activity effect for both younger and older participants during the performance of a VF task using fNIRS. Consequently, the findings of these last two studies show that both younger and older individuals may rely on neural compensation mechanisms as stated by the CRUNCH model.

## Growing of Life Differences Explains Normal Aging (GOLDEN Aging)

An assumption regarding normal aging that has not yet been mentioned in this review is that older adults tend to show increased distractibility compared to younger individuals. In other words, there is an age-related decline in top-down control due to diminished inhibitory function in the elderly, a model known as the reduced-inhibition hypothesis (Hasher and Zacks, 1988).

Based on the latter postulate, the reduced-inhibition hypothesis (Hasher and Zacks, 1988), and the CRUNCH model discussed earlier (Reuter-Lorenz and Cappell, 2008), Fabiani (2012) elaborated another theory on cognitive aging, known as the GOLDEN aging (Growing of life differences explains normal aging) model. This theory emphasizes that normal aging represents maturational processes causing progressive shifts in the distributions of mental abilities over the lifespan. More specifically, the GOLDEN aging model assumes that inhibitory processes are important to maintain representations as well as to determine the ability of working memory to process multiple strings of information both in the young and the elderly. Nonetheless, as postulated by the reduced-inhibition hypothesis, inhibitory processes tend to be weakened during lifespan development, leading to a reduction in the ability to maintain distinct representations in older adults (age-related decline in working memory performance). This being said, working memory capacity varies among individuals in the same age group and not all individuals show similar age-related declines in capacity. Consequently, it is the discrepancy between information load and working-memory capacity that leads any individual, young or old, to the recruitment of more and more cognitive resources until an asymptotic level is reached, as postulated by the CRUNCH model.

In summary, within the GOLDEN aging model, older individuals tend to show increased cerebral activation patterns such as bilateral recruiting of brain areas because of an age-related decrease in inhibitory processes (leading to a decrease in working memory capacity). However, even younger individuals can show similarly increased activation patterns during high-load cognitive processes.

## Temporal Hypothesis for Compensation (THC)

The previous compensatory mechanisms described so far presented anatomically based functional changes in brain activation patterns. However, there is still the possibility of another compensatory mechanism involving not so much WHICH regions of the brain show increased activation, but WHEN are these regions activated.

### Executive Function

Velanova et al. (2007) have suggested the existence of an age-related compensatory mechanism that consists of a shift from early to late selection processing during memory retrieval (the load-shift model). In fact, using the concepts of Rugg and Wilding (2000), who divided retrieval into three entities: retrieval orientation (anticipation of retrieval demands), retrieval effort (access of information), and postretrieval monitoring (evaluation of the appropriateness of the recollected information), Velanova et al. (2007) postulated that older participants would most probably rely more on retrieval effort and post-retrieval monitoring and less on retrieval orientation than younger individuals. To explore their hypothesis, they did two fMRI experiments. Thrity-six young and thirty-height old subjects participated in the first one, while twenty-nine young and thirty-seven old subjects were part of the second one. In both studies, participants had to distinguish new words from words that have been previously presented to them (old words), the difference being that in the first study there was only one type of load condition, while in the second experiment there were low (with old words repeatedly studied) and high load conditions (with old words only presented in the incidental deep encoding task, as for experiment 1). Data from both experiments showed that older adults had increased and delayed recruitment of frontal regions compared with the younger ones during demanding retrieval. Based on these results, the authors stated that this strategy shift could explain the retention of high-level cognitive function in some older individuals but at the expense of less flexible and slower performance on demanding cognitive tasks.

Paxton et al. (2008) contrasted the activity dynamics of younger and older adults during the performance of a cognitive control task (the Continuous Performance Test—AX version) relying on some executive processing (mainly discrimination ability and sustained attention). During the test, individuals are instructed to respond with a mouse press whenever the stimulus is an X that was preceded by an A. Their results showed a significant age-related temporal shift in lateral PFC regions: older adults presented both reduced cue-related (letter A) activation and increased probe-related (letter X) activation relative to younger adults. These findings are consistent with previous behavioral studies, in which older adults showed smaller cue-based expectancy effects but larger probe-related interference effects compared to younger individuals (Braver et al., 2001b, 2005; Paxton et al., 2006). Based on those results, Braver and colleagues (Braver et al., 2007) developed a theory, named dual mechanisms of control (DMC), which postulates a distinction between proactive and reactive modes of cognitive control. During the proactive control mode, individuals actively maintain in a sustained/anticipatory manner goal-relevant information before the occurrence of cognitively demanding events. On the other hand, in the reactive mode, attentional control is mobilized only when and if needed. Therefore, proactive control relies on the anticipation of interference before it occurs, while reactive control relies on the resolution of interference after its onset.

Jimura and Braver (2010) compared brain activity dynamics in healthy old and young adults during the switch and performance of two semantic classification tasks. During the first task, participants were required to make a decision as to whether a word described an object that is either larger or smaller than a computer monitor. During the second task, subjects had to make a decision as to whether the object was man-made or natural. Before every comparison (for both tasks), a cue appeared and signaled to the participants the semantic classification judgment to be made (LRG-SML or MAN-NAT). There were two block conditions in the study: the mixed-block condition during which the classification task to be performed varied randomly from trial to trial, and the single-task condition during which a single task was performed. Relative to young adults, older individuals presented decreased sustained activity in the anterior PFC during task-switching blocks, but increased transient activity on task-switch trials. Also, younger individuals showed a cue-related response during task-switch trials in the lateral PFC and posterior parietal cortex, whereas older adults presented switch-related activation during the cue period in posterior parietal cortex only. These results are in agreement with the DMC hypothesis and therefore suggest that older individuals shift from a proactive to reactive cognitive control strategy as a means of retaining relatively preserved behavioral performance despite age-related neurocognitive changes. It should be noted that this study is as much an executive function experiment (set-shifting) as it is a language processing experiment (semantic categorization).

**Figure 4:**
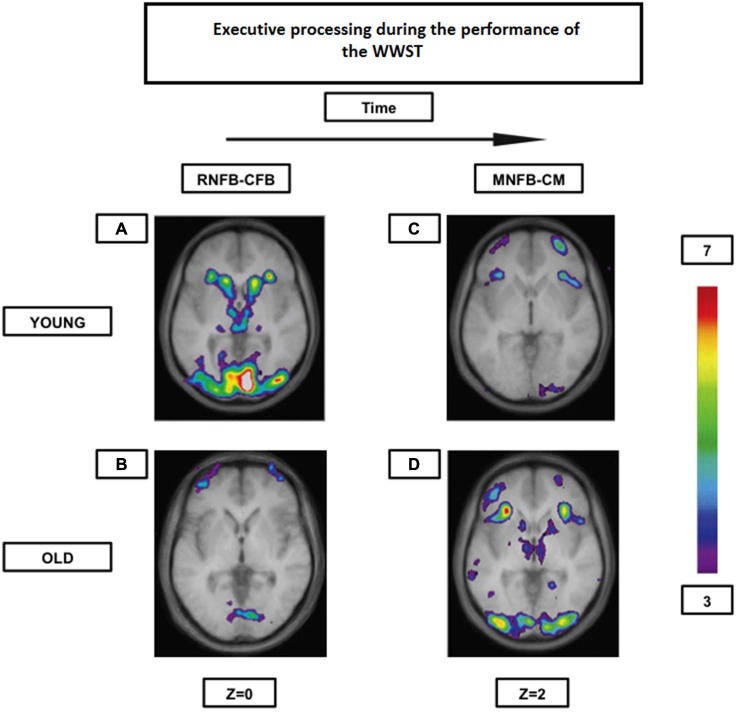
**Brain activation contrasts during executive processing using the WWST in young and old adults**. Old individuals recruit corticostriatal pathways later than young individuals during the WCST. **(A)**: Activation of a corticostriatal loop composed of the midventrolateral PFC (area 47/12), the caudate nucleus, and the thalamus when RNFB is compared to CFB in young adults. **(B)**: Activation of the frontopolar cortex (area 10) when RNFB is compared to CFB in old adults. **(C)**: Activation of the midventrolateral PFC (area 47/12) and the frontopolar cortex (area 10) when MNFB is compared to CM in old adults. **(D)**: Activation in a corticostriatal loop composed of the midventrolateral PFC (area 47/12), the caudate nucleus, and the thalamus, as well as in the putamen (which, with the posterior PFC, makes up another corticostriatal loop) when MNFB is compared to CM in old adults. The anatomical MRI images are the average of the T1 acquisitions of 14 younger subjects and 10 older participants transformed into stereotaxic space. The color scale represents the *T* statistic. RNFB: receiving negative feedback events; CFB: control feedback events; MNFB: Matching following negative feedback events; CM: Control matching **(A)** and **(B)** are reproduced from Figure 2 in Martins et al. (2012), and **(C)** and **(D)** are reproduced from Figure 3 in Martins et al. (2012).

Our group (Martins et al., 2012) has shown, as formerly mentioned, that both young and high performing older individuals seem to rely on the same frontostriatal loops while performing the WWST. However, whereas the young showed the involvement of a “cognitive loop” during the period that indicates that a set-shift will be required in the following trial (the receiving negative feedback period) and the involvement of a “motor loop” during the period when the set-shift must be performed (the matching after negative feedback period), the elderly showed significant activation of both loops during the matching after negative feedback period only (Figure 4). Thus, there seems to be an age-related shift in the timing of frontostriatal recruitment (delayed in the elderly). Consequently, those results may indicate, that with aging, individuals tend not to engage in costly executive processes until these become absolutely necessary. These results and this interpretation are also in agreement with the DMC hypothesis.

### Language Abilities

Cooke et al. (2006) performed an fMRI study on young adults in which they explored the neuroanatomic substrate and time course (using early and late time windows) associated with processing different grammatical features in a sentence. They used a grammatical test in which the participants had to judge the coherence of sentences that did or did not contain a grammatical violation. There were three possible violations: an inflectional form of the past participle (*ed* was omitted); a noun-verb substitution (rehearsed would be replaced by rehearsal for example); and a transitivity violation (a sentence containing a verb that cannot be expressed in a passive form because the verb is intransitive). These three violations are presented in an ascending order regarding cognitive demand. In early time windows, the participants showed significant left IFC recruitment in low-demanding judgments, and bilateral IFC recruitment in more-demanding judgments. In late time windows (BOLD activity levels measured 2 s later than the usual point at which the BOLD signal is monitored), the young participants did not show any activation during low-demanding conditions, but presented left IFC recruitment in the noun-verb substitutions and transitivity violations. Wingfield and Grossman (2006) presented, in their review article, that data with additional results from older individuals who performed that exact same task. The older participants showed a completely different pattern. Indeed, they increasingly activated the ventral portion of left IFC during the late time windows for even the simpler conditions; furthermore they also showed bilateral IFC activation during the more-demanding violations in the late time window (contrarily to unilateral activation for the young). Therefore, not only are those results compatible with the HAROLD model of neural compensation, they also show that the compensatory hypothesis may extend to the temporal domain for language processes.

### A New Hypothesis

In summary, age-related delayed brain region activation has been reported in several memory, executive, semantic categorisation and syntax processing experiments. Therefore, it does not appear to be a marginal finding, but a possible compensatory mechanism related to several cognitive domains and that may interact with other compensatory mechanisms such as neural compensation and neural reserve. Some of the experiments have shown that older individuals tend to present delayed activity in the frontal regions compared to younger individuals during tasks in which cognitive operations are not preceded by cues, such as in the studies of Velanova et al. (2007) and Wingfield and Grossman (2006). On the other hand, other studies from Braver and colleagues (Paxton et al., 2008; Jimura and Braver, 2010) as well as our group (Martins et al., 2012) have shown that in tasks during which cognitive operations are introduced by cues (anticipation phase) a shift in activity from the cue-phase to the probe-phase has been reported in older individuals. Those findings gave rise to the DMC hypothesis (Braver et al., 2007); with aging, individuals shift from proactive (anticipation) to reactive (resolution) cognitive control strategies. Our group (Martins et al., 2012) proposed that such an age-related shift could be beneficial since it allows for older individuals not to engage in costly cognitive processes until these become absolutely necessary.

**Figure 5:**
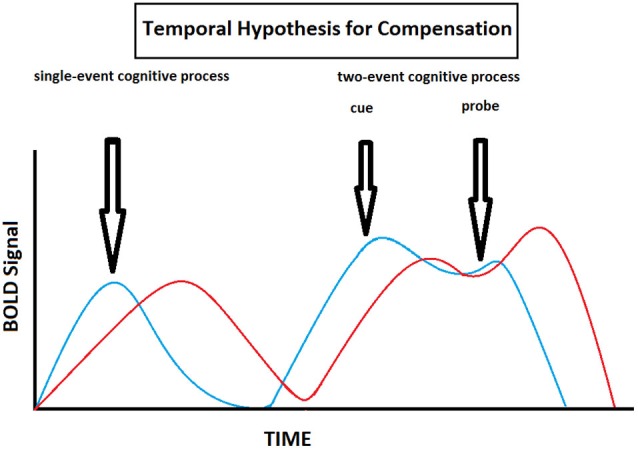
**Theoretical illustration of how BOLD signal varies with time in old (red) and young (blue) individuals according to the Temporal Hypothesis for Compensation**. This model implies that here is an age-related delay in brain activity during cognitive processing, and that old individuals shift from proactive to reactive cognitive control strategies compared to young individuals when cognitive processes imply both anticipation and resolution.

In this review, we propose to formalize all the age-related delayed brain region activation reported in the different studies under a single hypothesis, namely the Temporal Hypothesis for Compensation (THC). This hypothesis states the following: (1) there is an age-related delay in brain activity, particularly in the PFC, during cognitive processing (the PFC is singled out due to its primordial role in working memory); (2) there is an age-related shift from proactive to reactive cognitive control strategies when cognitive processes imply both anticipation and resolution; and (3) these age-related temporally based functional changes in brain activation patterns allow for cognitive performance to be preserved at the expense of speed processing (Table 2, Figure 5). This hypothesis complements and expends on the DMC model since it not only reiterates that older individuals tend to shift from proactive to reactive control strategies, but also show overall delayed activity in the PFC (and frontostriatal loops). This overall delay may be secondary to the fact that older individuals tend to operate information sequentially rather than concurrently due to diminished working memory capacity. By relying on reactive control strategies and sequential processing one might therefore be able to trade speed processing for cognitive performance even when there a discrepancy between demand and capacity (which tends to occur more often when one ages).

## Conclusion

As the passage of time structurally alters one’s brain, cognition does not have to suffer the same faith, at least not to the same extent. Indeed, the concept of CR coined by Stern (2002) implies that with age, decline in cognitive performance can be totally or partially compensated. Compensatory mechanisms can take the form of neural compensation and neural reserve. Neural compensation is the use of new, compensatory brain networks different from those pathways typically recruited for particular tasks (e.g., the HAROLD model, the PASA phenomenon). It should be noted that age-related over-activation may not always be compensatory, but may also represent dysfunction (the inability to inhibit certain brain areas) especially when it is correlated with impaired performance. Neural reserve, on the other hand, consists in using primary flexible brain networks or cognitive resources that are less susceptible to disruption.

It has been shown that compensatory mechanisms can also be used by young individuals when cognitive demands become significant. This observation led Reuter-Lorenz and Cappell (2008) and Reuter-Lorenz and Lustig (2005) to propose a model implying that people will generally activate more cortical regions as task load increases (the CRUNCH model). However, since older individuals might need to engage more neural resources at lower levels than younger adults (due to brain atrophy), it is expected for them to reach their resource limitations at lower levels of cognitive demand as well, leading to a decline in performance as demand increases. At this point, brain activity may plateau or even decrease with increasing task loads. A related hypothesis known as the GOLDEN aging model proposed by Fabiani (2012) postulates that older individuals show increased cerebral activation because of decreased inhibitory process ability associated with aging (leading to a decrease in working memory capacity). However, as for the CRUNCH model, younger individuals with substantial working memory capacity can also show similarly increased activation patterns during higher cognitive demand processes.

The previous compensatory mechanisms presented anatomically based functional changes in brain activation patterns. However, some studies have also shown delayed brain activation in older individuals compared to the young. Furthermore, those findings are associated with cognitive preservation. Therefore, we postulate that they represent another form of compensatory mechanism and we decided to formalize such a temporally based mechanism under the name of Temporal Hypothesis for Compensation (THC). This new theory builds on previous hypothesis that postulated a shift from proactive to reactive cognitive control strategies in the elderly, namely the DMC hypothesis.

In the introduction, we asked if different cognitive domains including language and executive function rely on similar compensatory mechanisms. Based on this review the elderly appear to present evidence of neural compensation, neural reserve and/or delayed brain activation (THC) while performing working memory, executive and/or language processes tasks. Why? First, because cognitive tasks rarely explore solely one cognitive domain at the time. Working memory, for example, not only merges with executive processes, but affects language abilities as well. It is known to constrain sentence comprehension especially regarding sentences with complex syntactic structures (Wingfield and Stine-Morrow, 2000) and plays a role in producing syntactically complex utterances (Kemper, 1992). Second, because there is a limited amount of strategies available: brain activation may be functionally reorganized from an anatomical perspective, from a temporal perspective, or from both perspectives. It makes intuitive sense for all cognitive domains to eventually rely on all these strategies so that performance can be maintained as demand increases.

However, as suggested by Ansado et al. (2013), the specific functional reorganization of the brain, that is which precise regions of the brain show increased or delayed activation for a given task, may take many different forms. For example, in language processing, while several studies looking at orthographic fluency have shown an age-related posterior-anterior shift in brain activation (PASA phenomenon), other studies exploring semantics presented an anterior-posterior shift instead. Both of these findings are examples of cognitive compensation, but the specifics of the compensation differ.

We believe that future research in the field of functional neuroimaging and aging (both normal and pathological) should try to explore the factors affecting these specific patterns of neurofuntional reorganization. Indeed, the nature of the task, the complexity of the task, the cognitive processes explored and the cognitive strategies used by participants appear to be determinant in the specific shape compensatory mechanisms will take. We also strongly believe that the THC model should be explored in greater depth given the little research there is on temporal neurofuntional reorganization compared to anatomical changes in activation patterns. Finally, there should be more functional connectivity studies whose role is to investigate age-related compensatory mechanisms since some studies suggest already that task-relevant functional connections between specific brain regions can be disrupted with age and that these disruptions have a negative impact on task performance (Madden et al., 2010; Bollinger et al., 2011).

## Conflict of Interest Statement

The authors declare that the research was conducted in the absence of any commercial or financial relationships that could be construed as a potential conflict of interest.
